# Rare Mimic of Conjunctivitis: Conjunctival Intraepithelial Sebaceous Carcinoma

**DOI:** 10.1155/crop/5565300

**Published:** 2025-09-19

**Authors:** Maedot A. Haymete, Kristen Delans, Paul Varghese, Nicholas Ramey, Douglas J. Grider

**Affiliations:** ^1^Virginia Tech Carilion School of Medicine, Virginia Polytechnic Institute and State University, Roanoke, Virginia, USA; ^2^Division of Dermatology, Department of Internal Medicine, Virginia Tech Carilion School of Medicine, Roanoke, Virginia, USA; ^3^Oculoplastics and Reconstructive Surgery, Vistar Eye Center Ophthalmology, Roanoke, Virginia, USA; ^4^Department of Basic Science Education, Virginia Tech Carilion School of Medicine, Virginia Polytechnic Institute and State University, Roanoke, Virginia, USA; ^5^Dominion Pathology Associates, Roanoke, Virginia, USA

## Abstract

Sebaceous carcinoma, an uncommon malignant neoplasm, often arises de novo from periocular sebaceous glands. Commonly manifesting as diffuse eyelid thickening, sebaceous carcinoma can mimic other inflammatory processes such as persistent chalazion or blepharitis. Delayed diagnosis often complicates the disease course due to its indolent presentation. Described is a rare case of sebaceous carcinoma entirely confined to the conjunctival epithelium of the upper eyelid. An 80-year-old female presented for evaluation of bothersome “cysts” under her left upper lid as well as blurry vision in her left eye. The patient was repeatedly re-evaluated over the next few months and found to have Meibomian gland dysfunction of the upper and lower left eyelids as well as 3+ diffuse spongy papillary injection of the tarsal conjunctiva in the left upper lid, with large papillae and conjunctival thickening in the left inferior fornix. A conjunctival biopsy was eventually performed when appropriate management of presumed conjunctivitis failed to alleviate the patient's symptoms. Pathological examination of the left upper eyelid tarsal conjunctiva showed epithelium largely replaced by pagetoid spread of intraepithelial sebaceous carcinoma, with an underlying band-like lymphocytic infiltrate. The carcinoma was strongly adipophilin positive with variable EMA, PRAME, and Factor XIIIa positivity, confirming intraepithelial sebaceous carcinoma. The nuclei of the carcinoma had a smudged, salt-and-pepper appearance; however, CK20, INSM-1, and synaptophysin were negative, excluding Merkel cell carcinoma. A sebaceous carcinoma limited to conjunctival epithelium is rare. However, given its potential aggressive nature, it should be included in the differential diagnosis of “nonhealing conjunctivitis” or persistent unilateral irritation of the eyelid.

## 1. Introduction

Sebaceous carcinoma is an uncommon malignant neoplasm arising from cells of sebaceous glands. It is often found in the eyelid and periorbital area [1]. Extraorbital sebaceous carcinoma can also occur in the head and neck regions, most commonly the parotid gland, and is less commonly noted in areas including the chest, extremities, and feet [1]. Sebaceous carcinoma of the eyelid often arises de novo from periocular sebaceous glands within the conjunctiva and the caruncle, Meibomian glands, or Zeis glands [2]. It commonly presents as diffuse eyelid thickening, with potential loss of cilia or a solitary eyelid nodule [2]. Sebaceous carcinoma of the eyelid may mimic an inflammatory process and is often misdiagnosed as persistent chalazion or blepharitis, leading to delays in management. Given its potential aggressive nature and risk of metastasis, it is especially important to consider sebaceous carcinoma in the differential diagnosis of elderly patients presenting with unilateral blepharitis [2]. Herein, presented is the case of an elderly female who presented with blurred vision of the left eye. She was initially treated for blepharoconjunctivitis, but after failure to improve with appropriate therapy, a biopsy was performed revealing pagetoid spread of sebaceous carcinoma confined to the conjunctival epithelium. The presentation of sebaceous carcinoma entirely limited to the epithelium is rare, and the case demonstrates an uncommon diagnosis that should be considered in the differential for patients with “nonhealing conjunctivitis” or persistent irritation of the eyelid.

## 2. Case Presentation

An 80-year-old female with a past ocular history of dermatochalasis and bilateral cataracts presented to an optometrist for evaluation of blurry vision in her left eye. Her past medical history included hypertension, hyperlipidemia, Type 2 diabetes mellitus, arthritis, asthma, bronchitis, irritable bowel syndrome, diverticulosis, and migraines. The patient complained of a “film covering the left eye,” occasionally affecting her vision at distance and near. She also reported bothersome foreign body sensation under her left upper eyelid and frequent episodes of eyelashes turning inward. She was diagnosed with chronic blepharoconjunctivitis and followed with no relief on topical steroid eye drops.

**Figure 1:**
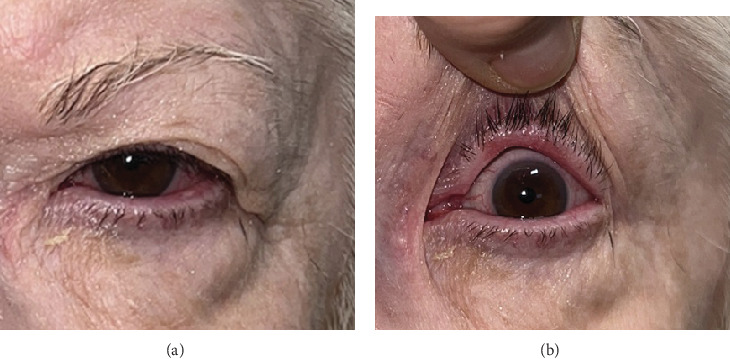
(a, b) The patient's left eye with red, injected conjunctiva.

**Figure 2:**
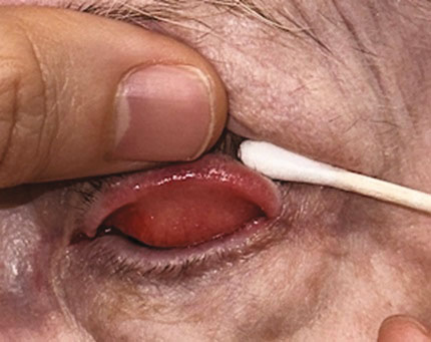
Bothersome “cysts” which on biopsy showed lymphoid hyperplasia of the left lower palpebral conjunctiva; clinical conjunctivitis mimicking sebaceous carcinoma confined to the left upper palpebral conjunctiva via pagetoid spread noted histopathologically.

At consultation with ophthalmology, she had 20/70 vision on the left, superficial punctate keratopathy, and limbal conjunctival injection. Trichiasis of the left upper eyelid was noted, in addition to 3+ diffuse spongy injection of the tarsal conjunctiva with large papillae and conjunctival thickening in the left inferior conjunctival fornix (Figures 1 and 2). No proptosis, dysmotility, or head and neck lymphadenopathy was present. Differential diagnosis included chronic ocular rosacea with blepharoconjunctivitis and sebaceous carcinoma. Incisional biopsies were performed on the palpebral conjunctiva of the left upper eyelid and the inferior conjunctival fornix of the left lower eyelid.

**Figure 3:**
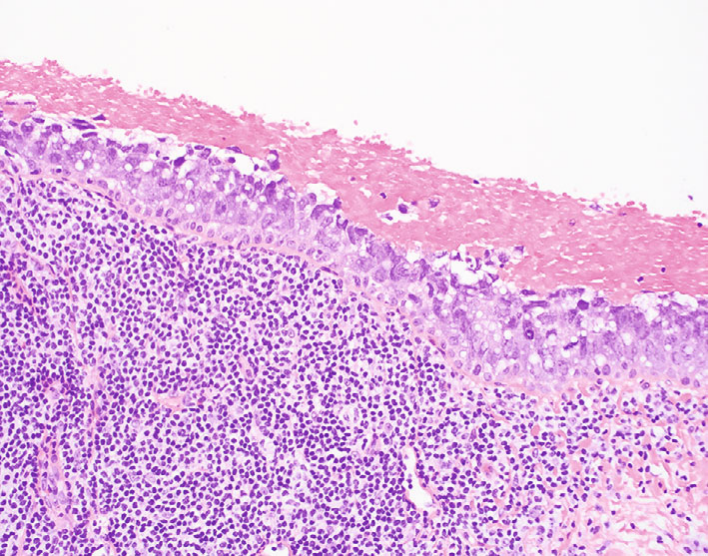
Conjunctival biopsy showing epithelium largely replaced by intraepidermal sebaceous carcinoma, with an underlying band-like lymphocytic infiltrate (H&E 20x).

**Figure 4:**
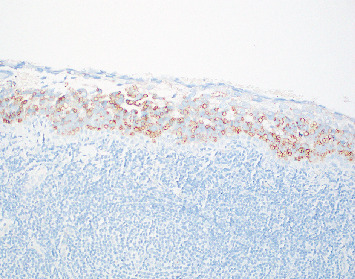
Adipophilin immunohistochemical study showing strong cytoplasmic positivity in the vacuolated intraepithelial malignant cells, confirming intraepithelial sebaceous carcinoma (20x).

**Figure 5:**
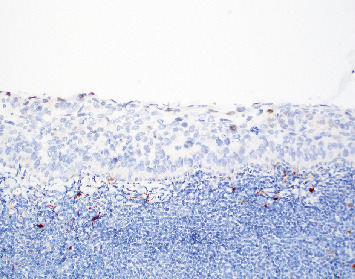
Factor XIIIa immunohistochemical study showing focal weak nuclear positivity in the intraepithelial malignant cells, supportive of intraepithelial sebaceous carcinoma (20x).

Biopsy of the left upper eyelid tarsal conjunctiva shows epithelium largely replaced by the pagetoid spread of intraepithelial cells with smudged, round nuclei and variable vacuolated cytoplasms, with an underlying band-like lymphocytic infiltrate (Figure 3). The intraepithelial cells in pagetoid array are adipophilin cytoplasmic positive (Figure 4). EMA and PRAME immunohistochemically stained tissue sections are focally cytoplasmic positive in the vacuolated cells and focally Factor XIIIa nuclear positive (Figure 5). The nuclei of the carcinoma have a somewhat smudged, salt-and-pepper appearance, as is seen with cells of neuroendocrine differentiation. However, CK20, synaptophysin, and INSM-1 are all negative, excluding Merkel cell carcinoma. Thus, the immunohistochemical profile and the histopathologic features noted support a diagnosis of intraepithelial sebaceous carcinoma. Additionally, mismatch repair protein studies were normal, helping to exclude Muir–Torre syndrome, as is often the case with periorbital sebaceous carcinomas.

Biopsy of the left inferior conjunctival fornix showed mucosa with several foci of lymphoid aggregates with germinal centers. The T-cell areas were positive for CD3 while the B-cell areas were positive for CD20, supporting reactive lymphoid hyperplasia. No sebaceous carcinoma were seen.

Treatment options, including palliative radiation, local resection, and subtotal orbital exenteration, were discussed with the patient. Surgery with frozen section analysis of margins or scout biopsies to determine the extent of the sebaceous carcinoma prior to surgery would aid in complete extirpation. The patient established care with oncology and a specialized ocular oncology service. However, she ultimately elected to pursue no further treatment.

## 3. Discussion

The eyelid and periorbital region harbors 5%–10% of all skin neoplasms, with basal cell carcinoma accounting for the vast majority of such malignancies [3]. Other less frequent neoplasms, such as sebaceous carcinoma, squamous cell carcinoma, and melanoma, have also been noted in the eyelid region [1]. Sebaceous carcinoma is primarily an ocular malignancy, commonly originating from the Meibomian glands and Zeis glands, as well as less frequent regions such as the eyebrow, conjunctiva, or multicenter origins [1].

Sebaceous carcinoma of the eyelid often arises de novo and has associated risk factors of advanced age, female sex, history of irradiation (for retinoblastoma or other conditions such as acne, cutaneous hemangioma, and eczema), immunosuppression (including HIV and HPV), and nitrosamine exposure (associated with diuretic use) [1]. Cutaneous sebaceous adenomas and, occasionally, periocular sebaceous carcinomas have been documented in patients with Muir–Torre syndrome, a form of mismatch repair protein deficiency [1]. Additionally, there is a higher incidence of sebaceous carcinoma in individuals of Caucasian and Asian backgrounds [4–8].

Sebaceous carcinoma may have one of several clinical presentations. Presentations noted in the literature include a unilateral solitary eyelid nodule, diffuse pseudoinflammation, pedunculated lesion, caruncular mass, eyebrow mass, lacrimal gland mass, or extensive orbital invasion [1]. Associated loss of cilia has also been noted. Sebaceous carcinoma is often referred to as “masquerading syndrome" because its clinical presentations are so varied and may mimic other conditions, which pose a diagnostic challenge. Sebaceous carcinoma is commonly misdiagnosed as chalazion, which is a product of noninfectious occlusion and subsequent inflammation of Meibomian glands [9]. However, unlike sebaceous carcinoma, chalazion is more commonly noted in younger patients, is often painful, and lacks diffuse involvement and loss of cilia. Sebaceous carcinoma may also be mistaken for blepharitis, conjunctivitis, and/or keratoconjunctivitis; however, these conditions are usually bilateral and do not cause notable thickening of the eyelids [1]. Additionally, sebaceous carcinoma may mimic other inflammatory conditions including unilateral papillary conjunctivitis, cicatricial pemphigoid, and sarcoidosis.

While sebaceous carcinoma of the eyelid is an infrequently reported diagnosis, sebaceous carcinoma entirely limited to the conjunctival epithelium of the eyelid via pagetoid spread is even more rare, with very few reports currently available in the literature [10–12]. In other more common presentations of sebaceous carcinoma, gross pathological samples often appear yellow due to the abundance of lipids. Four patterns are commonly noted on histopathology: (1) lobular type with less differentiated cells located in the periphery and more differentiated cells centrally; (2) comedocarcinoma type with a large, central necrotic core and viable cells in the periphery; (3) papillary type with papillary projections and sebaceous differentiation often in small conjunctival tumors; and (4) mixed type with patterns comprising the other three presentations [1]. Sebaceous carcinoma expresses EMA, Cam 5.2, and BRST-1 on immunohistochemistry. Sebaceous carcinoma can also be differentiated from its mimics through immunohistochemistry markers of adipophilin (cytoplasmic vacuolated positivity) and Factor XIIIa (nuclear positivity) [13].

There are several treatment options including local resection with or without frozen section evaluation of margin status and reconstruction, topical chemotherapy, cryotherapy, irradiation, and orbital exenteration for extensive spread [1]. Targeted therapies' immunotherapy may also be an option for eligible patients [14, 15]. Because of its invasive nature, orbital exenteration is the mainstay treatment for unresectable, aggressive sebaceous carcinoma. Hence, increased awareness of sebaceous carcinoma is vital for expedient identification and timely intervention.

## 4. Conclusion

Discussed is a rare case of sebaceous carcinoma of the eyelid confined to conjunctival epithelium by pagetoid spread associated with a significant delay in diagnosis due to an atypical presentation. Although sebaceous carcinoma usually presents as a tan-to-yellow nodule on the eyelid and a disease process limited to the conjunctival epithelium is exceedingly rare, its ability to “mimic” other conditions must be considered carefully in the appropriate patient. It is important to maintain sebaceous carcinoma on the differential for any patient who suffers from asymmetric, chronic blepharoconjunctivitis or chalazion for an extended period of time that is refractory to treatment.

## Data Availability

The data that support the findings of this study are available from the corresponding author upon reasonable request.
